# The G4 Resolvase DHX36 Possesses a Prognosis Significance and Exerts Tumour Suppressing Function Through Multiple Causal Regulations in Non-Small Cell Lung Cancer

**DOI:** 10.3389/fonc.2021.655757

**Published:** 2021-04-27

**Authors:** Yuxin Cui, Zhilei Li, Junxia Cao, Jane Lane, Emily Birkin, Xuefei Dong, Lijian Zhang, Wen G. Jiang

**Affiliations:** ^1^ Cardiff China Medical Research Collaborative, School of Medicine, Cardiff University, Cardiff, United Kingdom; ^2^ Department of Pharmacy, Zhujiang Hospital of Southern Medical University, Guangzhou, China; ^3^ Biotherapy Center, The Seventh Medical Center of PLA General Hospital, Beijing, China; ^4^ Cardiff & Vale University Health Board, University Hospital of Wales, Cardiff, United Kingdom; ^5^ Department of Thoracic Surgery, Peking University Cancer Hospital, Beijing, China

**Keywords:** DHX36, lung cancer, 3D spheroid, single-cell culture, drug, proteomics, transcriptomics

## Abstract

Lung cancer is one of the most prevalent cancers in both men and women worldwide. The nucleic acid G4 structures have been implicated in the transcriptional programmes of cancer-related genes in some cancers such as lung cancer. However, the role of the dominant G4 resolvase DHX36 in the progression of lung cancer remains unknown. In this study, by bioinformatic analysis of public datasets (TCGA and GEO), we find DHX36 is an independent prognosis indicator in non-small-cell lung carcinoma (NSCLC) with subtype dependence. The stable lentiviral knockdown of the DHX36 results in accelerated migration and aggregation of the S-phase subpopulation in lung cancer cells. The reduction of DHX36 level de-sensitises the proliferation response of lung cancer cells to chemotherapeutic drugs such as paclitaxel with cell dependence. The knockdown of this helicase leads to promoted tumour growth, demonstrated by a 3D fluorescence spheroid lung cancer model, and the stimulation of cell colony formation as shown by single-cell cultivation. High throughput proteomic array indicates that DHX36 functions in lung cancer cells through regulating multiple signalling pathways including activation of protein activity, protein autophosphorylation, Fc-receptor signalling pathway, response to peptide hormone and stress-activated protein kinase signalling cascade. A causal transcriptomic analysis suggests that DHX36 is significantly associated with mRNA surveillance, RNA degradation, DNA replication and Myc targets. Therefore, we unveil that DHX36 presents clinical significance and plays a role in tumour suppression in lung cancer, and propose a potentially new concept for an anti-cancer therapy based on helicase-specific targeting.

## Introduction

Lung cancer remains the most common cancer in both men and women worldwide, and the most common cause of cancer-related death (1). Despite the advances in research and patient care, only around 16% of lung cancer cases are diagnosed at an early stage and, for those patients with metastasis, the five-year survival rate is just 5% (2). Within all the unmet needs, the identification of new biomarkers for diagnosis, prognosis and treatment should still be addressed for the better care of lung cancer patients (3).

Guanine-quadruplex secondary structures (G4) are an architecture of nucleic acids that occur in guanine-rich sequences. They are four-stranded helical structures mediated by Hoogsteen hydrogen bonds formed between four guanines in a single plane, stabilised by a monovalent cation. G4s are alternative nucleic acid structures involved in essential nucleic acid metabolic processes including transcription, translation, RNA stability, DNA replication, DNA repair, and telomere maintenance (4–6). G4 structures function in gene transcription and post-transcriptional regulation of both coding and non-coding RNAs (7). G4s are enriched in gene promoter regions, regulatory regions of the human genome and untranslated regions of mRNAs (8–10). G4 motifs are present in the promoter regions of some proliferation-associated genes, proto-oncogenes, telomeres, repetitive DNA regions, and also in immunoglobulin heavy chain genes (11, 12). G4 structures at the promoters act as transcription repressors (13). Therefore, removing the G4 structure by replacing the critical guanine residues with adenines produces a several-fold increase in the transcription levels (14). G4 motifs in promoter regions may play a role in the regulation of genes involved in cell cycle regulation (13). G4 motifs in untranslated regions of some mRNAs can diminish their translation efficiency (15). G4s are found in the promoters of a wide range of cancer-related genes such as proto-oncogene MYC (14, 16), B cell lymphoma 2 (BCL-2) (17, 18), vascular endothelial growth factor (VEGF) (19), hypoxia-inducible factor 1α (HIF1α) (20), the transcription factor MYB (21), platelet-derived growth factor α polypeptide (PDGFA) (22), PDGF receptor β polypeptide (PDGFRβ) (23), KRAS (24–26), retinoblastoma protein 1 (RB1) (27, 28), mixed-lineage leukaemia (MLL) proto-oncogene (29) and human telomerase reverse transcriptase (TERT) (30). These genes are known to be implicated in cancer and are associated with the six hallmarks of cancer (31, 32).

The DEAH-box polypeptide 36 (DHX36) is also known as RNA helicase associated with the AU-rich RNA element (RHAU) or G4 resolvase-1 (G4R1). It is an ATP-dependent RNA helicase highly specific for DNA and RNA G4s (33, 34). The DEAH represents the code of the amino acids constituting motif II of the helicase domain (Asp-Glu-Ala-His) (35, 36). RNA helicases constitute a large group of essential enzymes that unwind or rearrange duplex and structured RNA molecules and function in a wide range of major RNA processing events (37–40). They are crucial to maintain genomic integrity and initiate the proper transcription and translation, by unwinding of nucleic acid secondary structures within cells. DHX36 can specifically bind and unwind the G4 motifs with its ATPase and resolving activity. DHX36 is considered as the major source of G4-DNA- and G4-RNA-resolving activities (41, 42). For example, DHX36 is found to play a critical role in the remodelling of G4s within the 5’-end of the human telomerase RNA. DHX36 resolution of telomeric RNA or DNA G4s activates telomerase for telomere elongation and increases RNA accumulation (43–45).

Structurally, DHX36 contains a conserved RNA helicase core region of approximately 440 amino acids, which consists of at least six discrete functional domains (46). The primary amino acid sequences of DHX36 consist of a region of the first 105 amino acids, which is termed as the RHAU-specific motif (RSM) and is essential and sufficient for direct RNA interactions, as well as its localization to stress granules in HeLa cells (47). A highly conserved 18 amino acid core sequence within this region is necessary for interaction with both RNA and DNA G4s (48). DHX36 has been initially found to be a critical factor in immunity. DHX36 functions as a DNA or double-stranded RNA (dsRNA) sensor in dendritic cells (49–51). It is a key molecule in RIG-I signalling that acts by forming an antiviral stress granule with dsRNA-dependent protein kinase (PKR), in a dsRNA-dependent manner, to execute antiviral reactions (47, 51, 52). Further, DHX36 plays a critical role in the interferon (IFN) signalling pathway by forming the DDX1-DDX21-DHX36 complex for dsRNA sensing (50).

The clinical significance and role of DHX36 in cancer are poorly understood, regardless of enormous indirect clues and a few highly specific studies. For example, DHX36 downregulates paired like homeodomain 1 (PITX1) while it upregulates Yin Yang 1 (YY1) transcription factor (53). These two genes both have G4 structures in their promotor regions and play diverse roles in modulating cancer progression. In response to UV-Induced DNA damage, DHX36 regulates the tumour suppressor p53 pre-mRNA 3’-end processing (54). Also, an RNA gene named G-Quadruplex Forming Sequence Containing LncRNA (GSEC) can bind to DHX36 *via* its G4 sequence, leading to the antagonisation of DHX36 and enhanced migration of colon cancer cells (55). In this study, we intended to evaluate the differential expression and prognostic value of DHX36 in patients with non-small-cell lung carcinoma (NSCLC). The role of DHX36 in lung cancer cells was also investigated using stable lung cancer cell models following DHX36 knockdown.

## Methods

### Clinical Significance Analysis

Overall survival analysis of DHX36 in lung cancer was conducted using the Kaplan-Meier plotter (https://kmplot.com). The probes of DHX36 were 223138_s_at, 223139_s_at and 223140_s_at. Patients were split by Auto select best cut-off. The size of eligible patients was 1144. Subtype breakdown meta-analysis of the survival analysis from different studies was performed using the web server of Lung Cancer Explorer (http://lce.biohpc.swmed.edu), which integrated the following eligible genomic data of GSE72094 (n=442) (56), TCGA (LUAD, n=576; LUSC, n=552) (https://www.cancer.gov/tcga), GSE30219 (n=307) (57), GSE41271 (n=275) (58), GSE31210 (n=224) (59), GSE31210 (224) (60), GSE37745 (n=196) (61), GSE50081 (n=181) (62), GSE42127 (n=176) (63), GSE11969 (n=163) (64), GSE19188 (n=156) (65), GSE13213 (n=117) (66), GSE3141(n=111) (67), GSE74777 (107) (68), GSE26939 (n=101) (69), GSE1037 (n=80) (70), GSE29016 (n=72) (71), GSE12472 (n=63) (72), GSE31548 (n=50) (73), GSE10245 (n=48) (74), GSE11117 (n=44) (75).

### Validation of DHX36 Expression in Human Lung Cancer Cohort From an In-House Lung Cancer Cohort

To verify the gene expression level of DHX36, in addition to the findings from the public available datasets, we carried out analyses of DHX36 expression on an existing lung cancer cohort of lung cancer patients with long-term follow-up from Peking University Cancer Hospital. Lung cancer and adjacent normal tissues were collected immediately after surgery and stored in liquid nitrogen until use. The study was approved by local ethics committees (Peking University Cancer Hospital) and performed in accordance with guidelines established by the World Medical Association Declaration of Helsinki. Written consent was obtained from all patients.

### Cell Lines and Culture Conditions

The lung cancer cell lines were purchased from the American Type Culture Collection (ATCC) and maintained at a low passage (less than 20). Cells were cultured at 37°C in a humidified incubator supplied with 5% CO_2_. Cells were grown in Dulbecco’s modified Eagle’s medium (DMEM; Sigma-Aldrich, Dorset, UK). Growth media for each cell line was supplemented with 10% foetal calf serum (FCS, PAA Laboratories Ltd., Somerset, UK), penicillin (100 U/ml), and streptomycin (100 mg/ml) (Sigma–Aldrich).

The A549 cell line is originally derived through explant cultivation of lung carcinomatous tissue from a 58-year-old Caucasian male, while SK-MES-1 is derived from the pleural effusion of a 65-year-old Caucasian male with lung squamous cell carcinoma. Both cell lines are hypotriploid in terms of their stemline chromosome number and possess relatively high frequencies of chromosome abnormality including number and structures. More details of the Karyotypes of these two cell lines can be found from ATCC (https://www.lgcstandards-atcc.org).

### Lentiviral Infection With DHX36 shRNA

Lentiviral vectors containing short hairpin RNAs (shRNA) specific for DHX36 and the control shRNA (Scr shRNA) were obtained from VectorBuilder (Santa Clara, CA, USA). The vectors were assembled with EGFP as a reporter and neomycin-resistant gene for selection. HEK293T packaging cells were transduced with viral packaging (psPAX2), viral envelope (pMD2G) and lentiviral plasmid vectors using serum-free OPTI-MEM (Invitrogen, Carlsbad, CA, USA) and FuGENE 6 transfection reagent (Promega, Southampton, UK). Four and five days after transfection, the supernatant containing the packaged viral particles was collected and filtered through a 0.45 µm filter. The cancer cells were then infected using the lentiviral supernatant in the presence of 8 μg/ml Polybrene (Sigma-Aldrich). After 48 hours, the cells were subjected to stable selection with 1.2 mg/ml G418 for 7 days, and maintained in a growth medium with 300 µg/ml G418. After selection, the stable lung cancer cell lines steadily expressed GFP, which could be visualised under a fluorescence microscope.

### Cell Proliferation Assay

Lung cancer cells at a density of 2,500 cells/well were seeded onto 96-well tissue culture plates. At designated time points, the cells were stained with Alamar Blue (Bio-Rad, Cambridge, MA, USA) following the manufacturer’s instruction. The fluorescence was read with an excitation wavelength of 530 nm and the emission at 590 nm using a Glomax Multi Detection System (Promega, Southampton, UK).

### Electric Cell-Substrate Impedance Sensing (ECIS)

The migration ability of the lung cancer cell lines was monitored using an ECIS system. Briefly, the lung cancer cells at a density of 2.5x10^4^ cell/well were seeded onto ECIS 96W1E array plates (Applied Biophysics Inc. NY, USA) and the electrical resistance, due to the interaction of cells and embedded gold-coated electrodes, was recorded. Once a confluent monolayer was formed, the cells were subjected to an electric wound at 2800 µA, 60 kHz for 20 s and the rate of change in impedance, as cells migrated onto the electrode sensing area, was subsequently monitored and analysed.

### Western Blotting

Cultured cells were washed twice in PBS and lysed in a RIPA buffer containing 50 mM Tris-HCl, 2% SDS, 5% glycerol, 5 mM EDTA, 1 mM NaF, 10 mM β-glycero-phosphate, 1 mM PMSF, 1 mM Na3VO4 and EDTA-free Protease Inhibitor Cocktail (Roche, Mannheim, Germany). Protein concentration was determined by the Pierce BCA protein assay (Thermo Scientific, Colchester, UK). After normalisation, proteins were separated by sodium dodecyl sulphate-polyacrylamide gel electrophoresis (SDS-PAGE) and transferred with a semi-dry fast transfer apparatus onto a PVDF membrane (Merck Millipore Inc., Billerica, USA). The membranes were blocked with 5% non-fat dried milk (Marvel, Premier Beverages, Stafford, UK) in PBST solution (0.05% Tween-20 in PBS) for 1 hour at room temperature. The membranes were then incubated with the primary antibodies diluted in 5% milk and left overnight at 4°C. The following day, they were washed in PBST and incubated with a diluted specific HRP-conjugated secondary antibody for 1 hour at room temperature. The primary antibody was DHX36 (diluted 1:1000. GTX131179, GeneTex, San Antonio, TX, USA) and β-actin (diluted 1:5000. sc 53142, Santa Cruz). The HRP-secondary antibodies (A5278, Anti-Mouse IgG; A0545, Anti-Rabbit IgG) were diluted at 1:2000 (Sigma-Aldrich, Dorset, UK). Protein detection was performed using an EZ-ECL chemiluminescence kit (Biological Industries USA, Inc., Cromwell, CT, USA). Immunoreactive bands were visualized and quantified by densitometry using the Syngene G: BOX chemiluminescence imaging system and Gene Tools 4.03 (Syngene Europe, Cambridge, UK).

### Reverse Transcription (RT) and Real-Time PCR Analysis

RNA was extracted from cultured cells at 60-80% confluency in T25 flasks or from fresh-frozen tissues using TRI Reagent (Sigma-Aldrich, Dorset, UK). Total RNA (500 ng) was reverse-transcribed to complementary DNA (cDNA) using Goscript Reverse Transcription mix (Promega). Following dilution of cDNA (1:8), quantitative real-time PCR was performed based on Amplifluor™ technology, in which a 6-carboxy-fluorescine-tagged Uniprimer™ (Biosearch Technologies, Inc., Petaluma, CA, USA.) was used as a probe, along with a pair of specific primers with an addition of a Z-sequence (actgaacctgaccgtaca) to the 5’-end of the reverse primer. The primer sequences for qPCR were: DHX36 forward primer, GTTTAAATCAGTTAACCAGACAC; DHX36 reverse primer, ACTGAACCTGACCGTACACGCAATGTTGGTAGCAATTA; β-actin forward primer, CATTAAGGAGAA GCTGTGCT; β-actin reverse primer, ACTGAACCTGACCGTACA GCTCGTAGCTCTTCTCCAG. The qPCR assays were run in a StepOnePlus system (Thermo Fisher Scientific, Waltham, MA, USA) and normalised by the corresponding threshold cycle (CT) values of β-actin mRNA.

### Flow Cytometry

Cultured cells were detached with trypsin/EDTA and fixed with the IC fixation buffer (ThermoFisher Scientific) for 1 hour at room temperature, then resuspended in ice-cold 100% methanol, and incubated overnight at -20°C. Cells were then washed twice in FACS buffer (2 mM EDTA in PBS, pH 7.4) and blocked with 1% bovine serum albumin (BSA) in PBS with 0.1% Tween for 1 hour. For the staining with antibodies, cells were incubated with diluted primary antibodies (1:100) including normal mouse IgG (14-4714-82, ThermoFisher Scientific) and cleaved poly (ADP-ribose) polymerase (PARP) (14-6668-82, ThermoFisher Scientific), respectively, for 1 hour at room temperature. Cells were then incubated with Alexa Fluor 647-conjugated goat anti-mouse IgG antibodies (1:1000; A21235, ThermoFisher Scientific) for 30 minutes at room temperature. For cell cycle analysis, cells were harvested and blocked as described above, and then directly incubated with Hoechst 33342 (10 µg/ml. H3570, ThermoFisher Scientific) for 1 hour at 37°C in the dark. Following the final wash with FACS buffer, FACS was performed using BD FACS Canto II flow cytometer equipped with FACS Diva Software (version 6.1.2. BD Biosciences, San Jose, CA, USA). FACS data were analysed using FCS Express software (version 4. De Novo Software, Los Angeles, CA, USA).

### Kaplan–Meier Survival Analysis

The association between DHX36 gene expression and the survival of the lung cancer patients was assessed using the pooled gene expression data from www.kmplot.com (Cut-off value: 1257.33. All probes included). The online tool allowed us to analyse both the OS (overall survival) and RFS (relapse-free survival) from 626 cases of lung cancer and the RFS from 1764 cases, which were subjected to expression profiling using Affymetrix GeneChip microarray (DHX36 Probeset ID: 223138_s_at; 223139_s_at; 223140_s_at).

### Kinexus Kinex Antibody Microarray

Cells were seeded in T75 flasks and incubated in DMEM supplemented with 10% FCS at 37°C. When the confluence was approximately 80%, the cells were washed twice and culture medium was replaced with DMEM containing 2% FCS. After incubation overnight, cells were suspended in lysis buffer, pH 7.4, containing 100mM Tris Buffer, 10% 2-ME, 1% NP-40, protease inhibitor cocktail tablet and 50 mM NaF. The lysates were vortexed and homogenised on a blood wheel for 1 hour at 4°C. The supernatant of the lysates was then collected by centrifugation for 30 minutes at 15,000 rpm at 4°C The protein concentration in the supernatant was determined by a fluorescamine protein quantification assay (Sigma-Aldrich). Proteomic analysis of pan-specific and phosphorylated proteins was carried out using high throughput Kinex antibody microarrays (900 antibodies, Kinexus Bioinformatics) (http://www.kinexus.ca/services).

### *In Vitro* 3D Tumour Spheroid Model

Monolayer cells, under exponential growth conditions, were resuspended at a density of 5000 cells/ml with 2% Matrigel (BD Biosciences, Oxford, UK). Cells were then seeded onto a 96-well Clear Polystyrene Cell-Repellent Microplate (#650970. Greiner bio-one, Germany) at a density of 500 cells/100 µl/well. Following overlay with 100 µl of the fresh normal medium, cells were cultured for 7 days to allow spheroid formation. The spheroids were then stained with propidium iodide (PI) at a working concentration of 1 µg/ml for 1 hour. The spheroid images were captured using the EVOS FL Auto 2 Imaging System (AMAFD2000. Fisher Scientific, Hemel Hempstead, UK).

### Bioinformatic Analysis

The code scripts for bioinformatic analysis were written in R version 3.4.4 (https://www.r-project.org) and Python version 3.9.0 (https://www.python.org). The Integrated Development Environment (IDE) for R was RStudio version 1.3.1093 (RStudio Inc), and the IDE for Python was the Jupyter Notebook version 6.1.5 (https://jupyter.org). The Bioconductor packages used in this study were Pathview (76), clusterProfiler (77) and org.Hs.eg.db version 3.2. 3 (78).

### Statistical Analysis

For quantitative measurement, including cell-based assays and gene expression profiling, the Shapiro-Wilk test was used to verify whether the data were normally distributed. Unpaired t-test was used for data with normal distributions whereas, for non-normal distributions, the Mann-Whitney Rank Test was applied. When more than two sets of data were compared, either One-Way ANOVA or the non-parametric Kruskal-Wallis test was used. Graphs and the statistical analysis were performed using GraphPad Prism version 8.4.3 (GraphPad Software, San Diego, CA, USA) or R (version 4.0.3, https://www.r-project.org). Statistical significance was indicated with the following nomenclature: *p<0.05, **p<0.01, ***p<0.001.

## Results

### Clinical Significance of DHX36 in NSCLC

**Figure 1:**
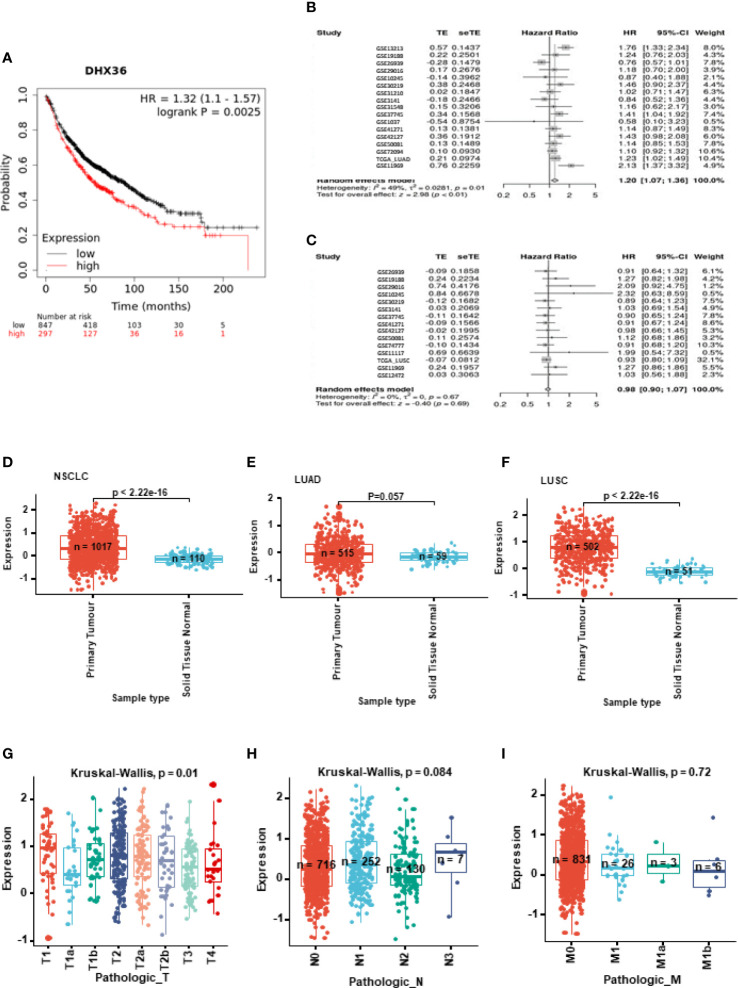
Clinical significance of DHX36 in lung cancer. **(A)** Overall survival analysis of DHX36 in lung cancer using the Kaplan-Meier plotter (https://kmplot.com). The probes of DHX36 were 223138_s_at, 223139_s_at and 223140_s_at. The patients were split by Auto select best cut-off. The number of eligible patients was n=1144. The breakdown meta-analysis of survival analysis in LUAD **(B)** and LUSC **(C)** was performed using the Lung Cancer Explorer (http://lce.biohpc.swmed.edu), which integrated the following eligible genomic data of GSE72094 (n=442), TCGA (LUAD, n=576; LUSC, n=552), GSE30219 (n=307), GSE41271 (n=275), GSE31210 (n=224), GSE31210 (224), GSE37745 (n=196), GSE50081 (n=181), GSE42127 (n=176), GSE11969 (n=163), GSE19188 (n=156), GSE13213 (n=117), GSE3141 (n=111), GSE74777 (107), GSE26939 (n=101), GSE1037 (n=80), GSE29016 (n=72), GSE12472 (n=63), GSE31548 (n=50), GSE10245 (n=48) and GSE11117 (44). **(D)** Differential expression of DHX36 gene between tumour and normal tissues in NSCLC. **(E)** Differential expression of DHX36 gene between tumour and normal tissues in LUAD. **(F)** Differential expression of DHX36 gene between tumour and normal tissues in LUSC. **(G)** Differential expression of DHX36 gene among T staging types in NSCLC. **(H)** Differential expression of DHX36 gene among N staging types in NSCLC. **(I)** Differential expression of DHX36 gene among M staging types in NSCLC.

KM plotting analysis indicated high DHX36 gene expression was associated with poor overall survival of lung cancer patients (HR=1.32, logrank p=0.0025, n=1144; **Figure 1A**). We further conducted the breakdown meta-analysis of DHX36 gene expression using all the publicly available transcriptome studies. As shown in **Figure 1B**, in LUAD, the HR for random effect models was 1.20, indicating that the high level of DHX36 gene expression was associated with poor overall survival (p<0.01). In LUSC (**Figure 1C**), the HR for random effect models was 0.98, and the gene expression of DHX36 was not significantly associated with patient survival (p=0.69), suggesting there was subtype-dependence.

In all NSCLC samples, gene expression of DHX36 was higher in the tumour group (n=1017) than in normal tissues (n=110) (p<0.0001; **Figure 1D**). In the LUAD subtype, there was a significant difference in the DHX36 gene expression level between tumour (n=515) and normal tissue (n=59) groups (**Figure 1E**). In the LUSC subset, gene expression of DHX36 was higher in the tumour group (n=502) than in normal tissues (n=51) (p<0.0001; **Figure 1F**), which was similar to the pattern before subtype classification. We also compared the gene expression of DHX36 among different Tumour-Node-Metastasis (TNM) staging types. The different T stages were significant (p=0.01), however, it was not possible to outline the definitive trends given the wide variation (**Figure 1G**). There was no significance observed among the different N (**Figure 1H**) and M stages (**Figure 1I**). It might be important to note that the limited sample size at certain stages might make the statistical analysis lack power.

We verified the differential gene expression of DHX36 in lung cancer using our in-house cohort by qRT-PCR. It showed that there was a lower expression of the DXH36 gene in the tumour tissue (p<0.05) despite wide variation (**Supplementary Table 1**).

### Establishment of Stable Lung Cancer Cell Lines With DHX36 Knockdown (KD)

**Figure 2:**
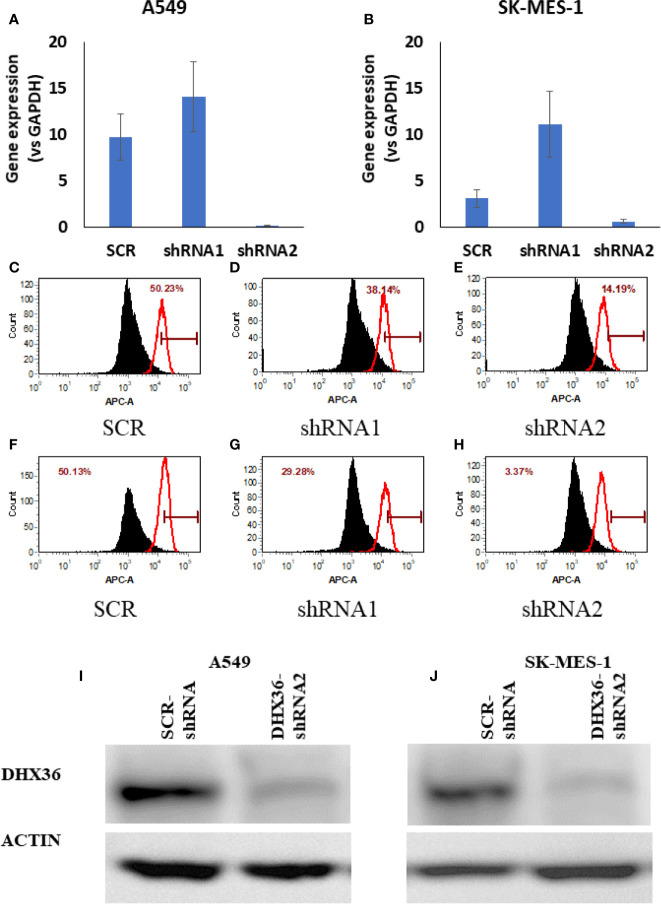
Validation of the stable cell lines with DHX36 shRNAs. Gene expression in A549 **(A)** and SK-MES-1 **(B)**. Flow cytometry analysis of DHX36 protein expression in A549 containing SCR **(C)**, A549 containing DHX36 shRNA1 **(D)**, A549 containing DHX36 shRNA2 **(E)**, SK-MES-1 containing SCR **(F)**, SK-MES-1 containing DHX36 shRNA1 **(G)**, SK-MES-1 containing DHX36 shRNA1 **(H)**. Western blots of DHX36 protein in A549 **(I)** and SK-MES-1 **(J)**.

Given the discrepant association of DHX36 with prognosis and clinicopathological features, we next attempted to determine the role of DHX36 in NSCLC. We established stable DHX36-KD cell lines using two lung cancer cells, A549 and SK-MES-1. As shown in **Figures 2A, B**, the qRT-PCR data showed that the gene expression level of DHX36 was dramatically decreased by DHX36-shRNA2 but not DHX36-shRNA1 compared to the SCR control. The FACS data indicated that in A549 cells the protein level of DHX36 decreased by 24% in the shRNA1 group, but this level was reduced by 72% in the shRNA2 one (**Figures 2C–E**). Similarly, in SK-MES-1 cells, the protein level of DHX36 decreased by 42% in the shRNA1 group, while this level was reduced by 93% by shRNA2 (**Figures 2F–H**). We then used the cell lines containing shRNA to validate the change of protein level using Western blot. As shown in **Figures 2I, J**, the reduction of DHX36 by shRNA2 was further confirmed in both parental cell lines. We, therefore, established the stable cell lines using the shRNA2 and labelled them KD.

### Knockdown of DHX36 in Lung Cancer Cells Enhanced Migration

**Figure 3:**
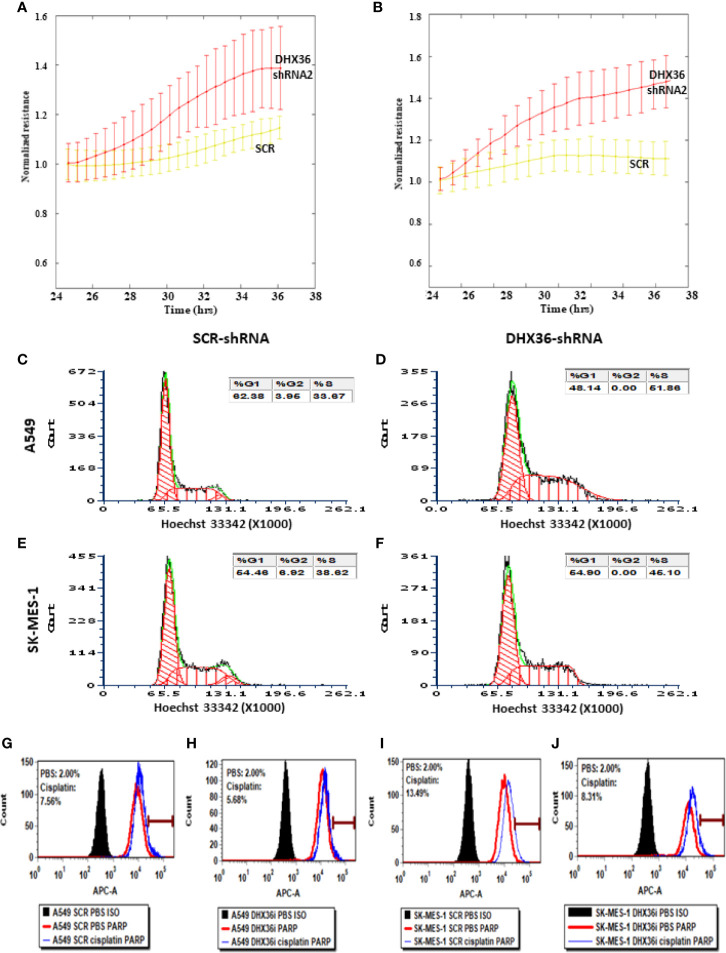
The effect of the knockdown of the DHX36 gene on cell migration, cell cycle and apoptosis. The migration was monitored by the ECIS system in the stable cell lines derived from A549 **(A)** and SK-MES-1 **(B)**. **(C)** Cell cycle analysis of A549 SCR. **(D)** Cell cycle analysis of A549 DHX36 KD. **(E)** Cell cycle analysis of SK-MES-1 SCR. **(F)** Cell cycle analysis of SK-MES-1 KD. **(G)** Apoptosis of the A549 SCR treated with PBS. **(H)** Apoptosis of the A549 DHX36 KD treated with cisplatin (10 µM, 24 h). **(I)** Apoptosis of the SK-MES-1 DHX36 KD treated with PBS. **(J)** Apoptosis of the SK-MES-1 DHX36 KD treated with cisplatin.

We monitored the real-time migration of stable cell lines using the ECIS system. In both A549 and SK-MES-1 cells, the knockdown of DHX36 significantly promoted cell migration (p<0.05 vs SCR, respectively; **Figures 3A, B**). We also determined the cell cycle phase distribution by FACS. As shown in **Figures 3C, D**, in A549 cells the S-phase populations increased from 33.67% in the SCR control to 51.86% in the DHX36-shRNA. Similarly, in SK-MES-1 cells the S-phase populations increased from 38.62% in the SCR control to 45.10% in the DHX36 KD group (**Figures 3E, F**). The increase of the S-phase indicated the promotion of cell division. We then investigated the apoptosis of cells in response to the cytotoxic drug cisplatin. In the four stable cell lines, we observed an increase in cisplatin-induced apoptosis. However, there was no significant difference in the cisplatin-induced increase among the different cell lines, suggesting DHX36 may not be involved in apoptosis induced by cisplatin (**Figures 3G–J**).

### Knockdown of DHX36 Promoted Tumour Growth in a 3D Culture Environment

**Figure 4:**
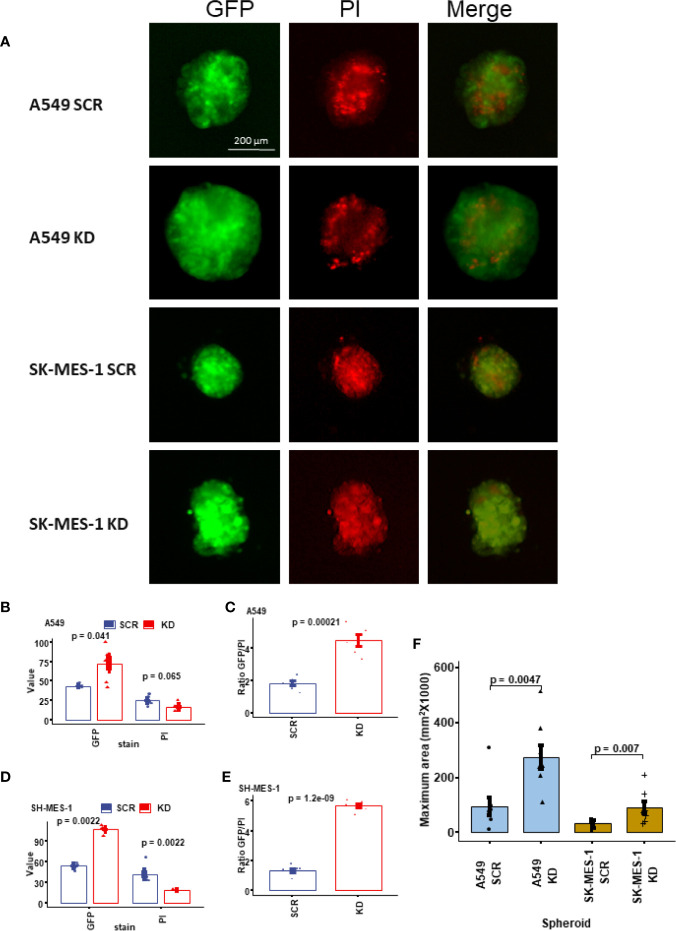
Growth and viability analysis of the 3D tumour spheroids. **(A)** Representative images of the lung cancer cell spheroids. The spheroids were stained with propidium iodide (PI) to indicate cell death. **(B)** The value of GFP and PI in the stable A549 cell lines. **(C)** The ratio of GFP/PI in the stable A549 cell lines indicating the viability level. **(D)** The value of GFP and PI in the stable SK-MES-1 cell lines. **(E)** The ratio of GFP/PI in the stable SK-MES-1 cell lines. **(F)** Comparison of the sizes (indicated by the maximum area) of the spheroids.

We fabricated 3D lung cell spheroids to understand the tumour growth and change of viability mediated by the knockdown of DHX36 (**Figure 4A**). As shown in **Figure 4B**, the knockdown of DHX36 increased the integrated median density of GFP, which is used to quantify the overall cell number compared to the SCR control in A549 cells (p=0.041). There was also a trend of decreased PI intensity, which discriminates dead cells (p>0.05). The ratio of GFP/PI indicated that the DHX36 knockdown increased the viability of A549 cells dramatically (p=0.00021; **Figure 4C**). Likewise, the knockdown of DHX36 in SK-MES-1 cells increased the GFP intensity (p=0.0022), decreased PI intensity (p=0.0022) and in turn increased the GFP/PI (p<0.0001) in the spheroids (**Figures 4D, E**). The data, therefore, indicated that the knockdown of DHX36 enhanced the viability of lung cancer cells in 3D spheroid culture. We then compared the size of spheroids by measuring the area of the maximum cross sections of individual spheroids. This showed that the knockdown of DHX36 led to an increase in spheroid size in both A549 and SK-MES-1 cells (p<0.01 vs SCR, respectively; **Figure 4F**).

### Knockdown of DHX36 Promoted the Colony Formation of Single-Cell Culture

**Figure 5:**
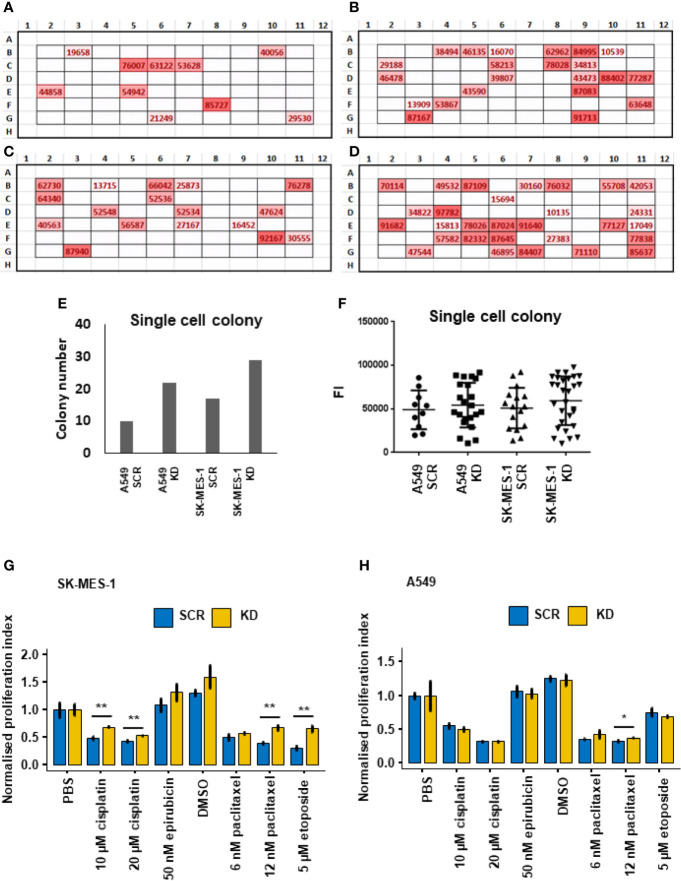
Single-cell colony formation and cellular response to cytotoxic drugs. **(A)** *In situ* heatmap of the single-cell colony formation from the A549 SCR cell line in a 96-well plate following cultivation for 3 weeks. The values are fluorescent intensity (FI) indicated by the Alamar Blue proliferation assay. **(B)** *In situ* heatmap of the single-cell colony formation from the A549 KD cell line. **(C)** *In situ* heatmap of the single-cell colony formation from the SK-MES-1 SCR cell line. **(D)** *In situ* heatmap of the single-cell colony formation from the SK-MES-1 KD cell line. **(E)** Quantification of the colony numbers following single-cell culture. **(F)** Analysis of the FI values of the colonies indicated by Alamar Blue. **(G)** Cytotoxic response of the stable A549 cell lines to chemotherapeutic drugs indicated by Alamar Blue. The normalised proliferation index is calculated by the division of the FI value of each group with the PBS group. **(H)** Cytotoxic response of the stable SK-MES-1 cell lines to chemotherapeutic drugs. *p < 0.05, **p < 0.01.

To understand how single cells proliferate, we performed single-cell culture in 96 well plates. After single-cell culture for 3 weeks, there was a significant increase in colony numbers in the DHX36 shRNA knockdown groups in both A549 (**Figures 5A, B**) and SK-MES-1 cells (**Figures 5C, D**). The increase in colony numbers was approximately 2.2 fold in the A549 cells and 1.7 fold in SK-MES-1 cells (**Figure 5E**). We quantified the relative cell number of each colony using the Alamar Blue assay, and found no difference among the groups, suggesting there was no difference of cell number per colony in each cell population (**Figure 5F**).

### Effect of DHX36 Knockdown on Cell Sensitivity to Cytotoxic Drugs

We then evaluated the response of the lung cancer cells to chemotherapeutic drugs including cisplatin, epirubicin, paclitaxel and etoposide. Overall, in all the stable cell lines derived from SK-MES-1 and A549 cells, the four selected drugs reduced cell proliferation levels dramatically in the cells with both DHX36 knockdown and SCR control. In the stable SK-MES-1 cells (**Figure 5G**), DHX36 knockdown showed a higher remaining proliferation level compared to the SCR control in cells treated with cisplatin at concentrations of 10µM and 20µM (both p<0.01). There was no response to 50nM epirubicin between the two cell stable cell lines. Also, in cells derived from SK-MES-1, DHX36 knockdown showed a higher proliferation level in response to 12nM paclitaxel compared to the SCR control (p<0.01), which was not observed in the treatments with 6 nM paclitaxel. Likewise, DHX36 knockdown in the SK-MES-1 cells led to a higher remaining proliferation level compared to the SCR control after treatment with 5 µM etoposide (p<0.01). In the stable A549 cells (**Figure 5H**), a significant difference of the proliferation levels between the DHX36 group and its SCR control could only be observed following treatment with 12 nM paclitaxel (p<0.05).

### Proteomic Analysis Indicated the Change of Signalling Pathways After the Knockdown of DHX36

**Figure 6:**
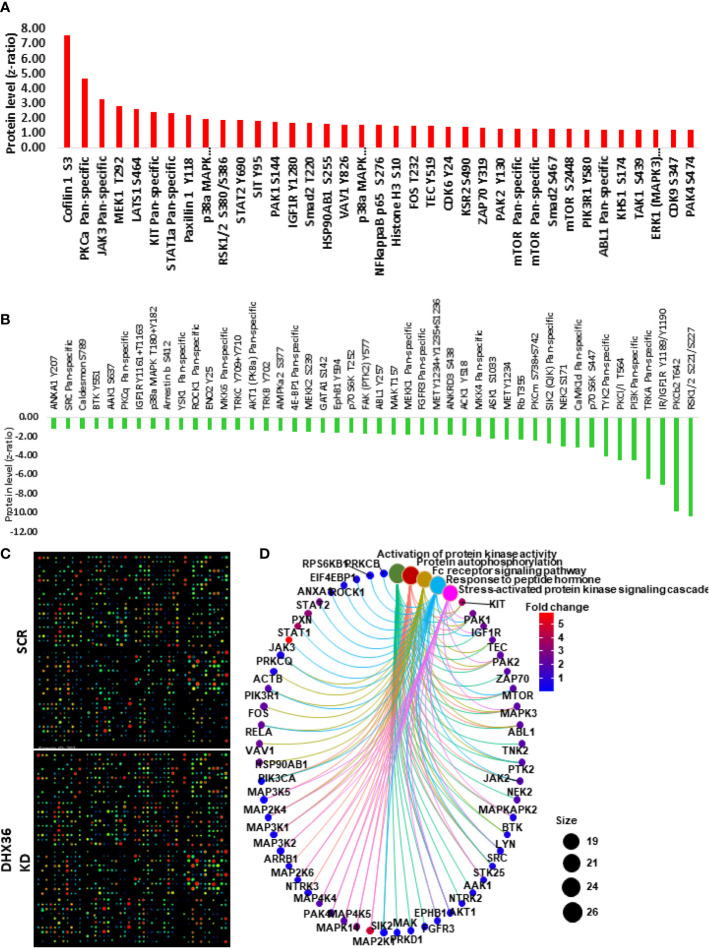
Kinex antibody microarray analysis of signal transduction after DHX36 was knocked down in A549 cells. **(A)** Upregulated proteins with z ratio>1 **(B)** Downregulated proteins with z ratio<-1 **(C)** Heatmaps of the Kinex antibody arrays. **(D)** Key signalling pathways identified using the clusterProfiler Bioconductor package in R language.

We conducted the Kinex antibody array to understand whether the knockdown of DHX36 could alter the signalling profile of the stable A549 cell lines. The upregulated proteins are outlined in **Figure 6A**, while the downregulated proteins are outlined in **Figure 6B**. The heatmap of the antibody arrays is shown in **Figure 6C**. To depict the linkages of the altered proteins and the most significant biological functions in a signalling complex network, we conducted protein enrichment plotting using a Bioconductor package of genome-wide annotation for human (org.Hs.eg.db). As shown in **Figure 6D**, the most significant biological functions that DHX36 regulated were activation of protein activity, protein autophosphorylation, Fc receptor signalling pathway, response to peptide hormone and stress-activated protein kinase signalling cascade.

### Differential Expression Analysis Unveiled the Role of DHX36 in Gene Regulation in Lung Cancer

**Figure 7:**
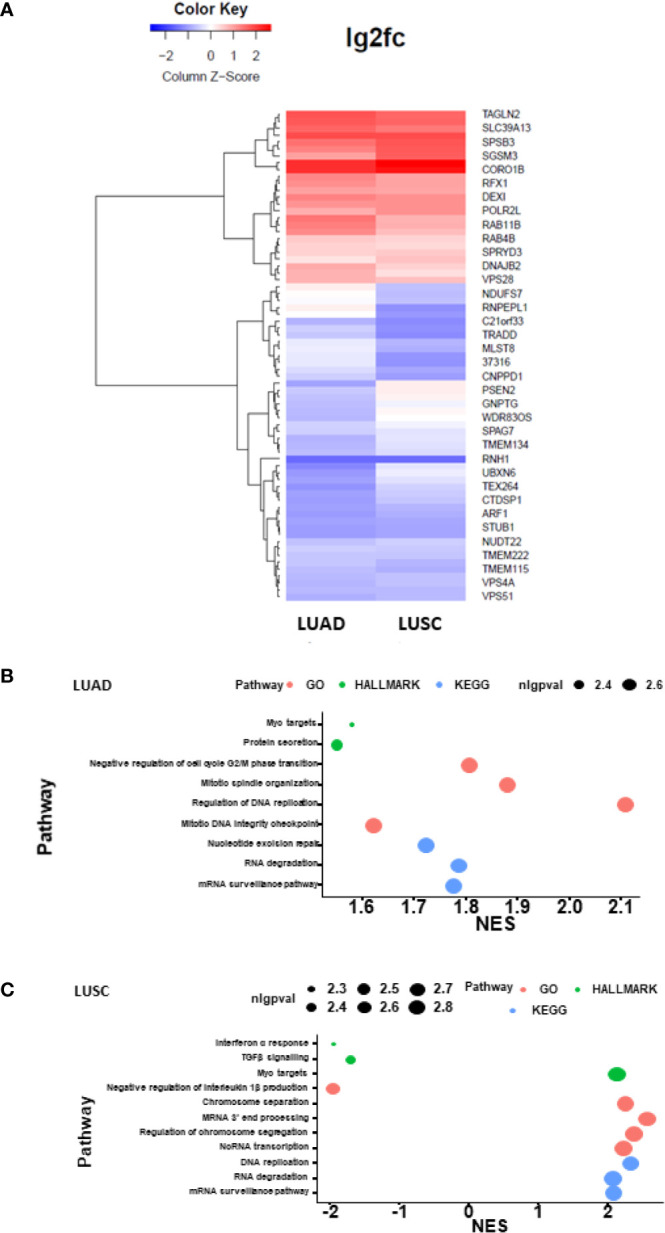
Heatmap plotting and causal pathway analysis of the differential transcript profiles affected by DHX36 in LUAD and LUSC. The bioinformatic analysis was conducted using the Pathview Bioconductor package in R. The lung cancer RNA-Seq datasets were obtained from TCGA. **(A)** Heatmap visualisation of the differential transcripts affected by DHX36. **(B)** Causal gene set enrichment analysis in LUAD. **(C)** Causal gene set enrichment analysis in LUSC. The enrichment scores were obtained using multiplex pathway databases including Gene ontology terms (GO), Kyoto Encyclopedia of Genes and Genomes (KEGG) and the Molecular Signatures Database (MSigDB) HALLMARK sets. nlgpval is the abbreviation of the negative log of p value.

We performed bioinformatic analysis of the TCGA RNA-Seq datasets of lung cancer in order to identify the causal regulation pathway signature. The heatmap plotting of the DHX36-associated differential genes in LUAD and LUSC showed that DHX36 had different transcript profiles while sharing certain levels of similarity (**Figure 7A**). As a result of the gene set enrichment analysis, DHX36 was associated with mRNA surveillance, RNA degradation, DNA replication and Myc targets in both subtypes (**Figures 7B, C**). In LUAD, DHX36 was also associated with protein secretion, cell cycle G2/M transition, mitotic spindle organization, and nucleotide excision repair. In LUSC, DHX36 was also associated with interferon α response, TGFβ signalling, negative regulation of interleukin 1β production, chromosome separation, mRNA 3’-end processing, regulation of chromosome segregation, and ncRNA transcription.

## Discussion

There is no previous report of the clinical significance of DHX36 in lung cancer. We show that the gene expression level of DHX36 is negatively associated with patients with NSCLC. The meta-analysis of all the publicly available datasets from previous studies indicates the negative association exists in LUAD instead of in LUSC. However, our differential gene expression analysis indicates that in both LUSC and LUAD subtypes, the levels of DHX36 gene expression in lung cancer are higher than in normal tissues. Therefore DHX36 indeed plays a role in indicating malignancy and subtype-dependent patient survival. An analysis of the clinical significance in pan cancers using a web server GEPIA (http://gepia2.cancer-pku.cn) further confirms that DHX36 gene expression is increased in lung cancer but is varied in other cancers (**Supplementary Figure 1A**). NSCLC is the cancer type with the second-highest frequency of altered DHX36 amplification, indicated by the cBioPortal for Cancer Genomics (https://www.cbioportal.org) (**Supplementary Figure 1B**).

Although the TCGA dataset shows higher expression of DHX36 gene in tumour tissue, which is in line with the altered protein levels in a CPTAC dataset, there are also other studies showing that the transcript level of DHX36 is lower in the tumour samples (**Supplementary Figure 2**). These discrepancies may occur due to the sampling method, low numbers of normal controls, tumour tissue heterogeneity or normalisation algorithms. Future studies, with large-scale high-quality samples and techniques with high sensitivity and accuracy, may enable us to make a robust conclusion.

Using the validated shRNA we can establish stable lung cancer cell lines with DHX36 knockdown. We demonstrate that the knockdown of DHX36 enhances the migration capacity of lung cancer cells. Whilst there is no previous report of DHX36 function in lung cancer, there is indirect evidence with DHX9, a DHX36 paralog among the DEAH-box helicases. DHX9 and DHX36 are the only two helicase enzymes that have been reported to unwind both DNA and RNA G-quadruplexes (41, 79). These two helicases share the domains of the DEAH motif, a domain of the unknown function (DUF), helicase-associated domain 2 (HA2) and helicase C-terminal domain (49). It is reported that the knockdown of DHX9 can promote the migration of A549 cells, suggesting they may have a similarity in the role of mediating the migration of cancer cells through a shared functional domain (80). For potential mechanisms, our protein array data show that after the knockdown of DHX36, there is a significant increase of migration driver Protein Kinase C α (PKCA) in the stable cell line. PKCA is considered to repress the transcription of p120-catenin, leading to the destabilisation of E-cadherin and the dissociation of adhesion junctions, therefore promoting cell migration (81).

We show that there is an accumulation of S-phase cell population after DHX36 knockdown, suggesting that DHX36 plays a role in modulating DNA replication and cell cycle progression. There is experimental evidence that DHX36 increases the translational efficiency of its targets through harbouring and unwinding the nucleic acid G4 structures (82). The cell cycle is regulated by various cyclin-dependent kinases (CDKs), a group of serine/threonine-specific protein kinases with approximately 20 members (83). The formation of the CDK-cyclin complex enhances the accumulation of the prospective phase in a cell cycle. Our Kinex antibody array analysis indicates that the phosphorylation levels of CDK6 and CDK9 are increased in response to the DHX36 knockdown. CDK6 is known to form a complex with cyclin D and drive the cell-cycle progression from the G0/G1 phase into the S phase (84). CDK9 plays a regulatory role in the transcription of cyclins such as upregulating cyclin D but downregulating cyclin E, thus is vital in the completion of DNA synthesis in response to replication stress (85).

Cancer cells can be less quiescent when there is an accumulation of S-phase population, hence may be more susceptible to chemotherapeutic drugs. We show, however, that lung cancer cells exhibit less or similar cytotoxicity in response to therapeutic drugs including cisplatin, epirubicin, paclitaxel and etoposide. This implies that the drug response in lung cancer cells can be varied and may depend on multiple factors such as cell division, metabolism, uptake and targeting mechanisms (86).

High throughput proteomics may better identify the altered signalling pathways in cancer cells after gene knockdown. Our high throughput antibody analysis indicates that, after the knockdown of the DHX36 gene in stable cell lines, key cellular signalling transduction occurs from protein kinase activation, protein phosphorylation, Fc receptor signalling, response to peptide hormone and stress-activated protein kinases. This demonstrates that DHX36 is indeed a multifunctional protein and its role in the stress process in tumour cells.

We have attempted to explore the causal regulator of the DHX36 function by taking advantage of the TCGA datasets. There is a certain similarity of the differential gene expression profile associated with DHX36 levels between two NSCLC subtypes. For causal pathways highlighted, mRNA surveillance functions through quality control to induce the degradation of aberrant mRNA thus preventing its translation to cytotoxic proteins. Although the role of mRNA surveillance in cancer can be complicated, there may be potential to target this pathway and allow the formation of truncated proteins with loss of function. DHX36 has also been reported to resolve the G4s which are located in either the 5’-UTR or the 3-UTR of mRNAs and modulate their degradation (87). In addition, the relatively stable folded G4s in the single-stranded DNA can be disrupted by DHX36 in an ATP-dependent manner, which therefore regulates the position of the DNA forks in replication, and may shift genetic instability caused by aberrant DNA G4s. The complex of DHX36-specific motif (DSM) and the G4-enriched c-Myc promoter sequence has been unveiled by crystal structure modelling (46). It is therefore understandable that DHX36 plays a role in regulating the pathway of the Myc targets. It is known that as a proto-oncogene, Myc drives downstream transcription amplification thus leading to augmented tumour growth. In contrast, our data suggest that DHX36 is a tumour suppressor based on a variety of cellular assays. We, therefore, speculate that DHX36 is multifunctional but may not be predominantly through the Myc target activity.

Our data indicate that DHX36 is a novel independent prognosis indicator in NSCLC with subtype dependence using both publicly available large-scale datasets and our in-house cohort. The stable lentiviral knockdown of the DHX36 allows us to determine the role of DHX36 as a tumour suppressor through a variety of cellular and molecular assays including migration, ECIS, 3D tumour model and single-cell colony formation. Interestingly, we also identify the effect of DHX36 on tumour cell susceptibility to chemotherapeutic drugs, which imply its therapeutic potential. Given the nature that DHX36 is a multifunctional G4-structure resolvase, that may influence the multiple steps of RNA metabolism, we explore transcriptomics and proteomics in systematic ways to outline the critical regulation, alteration and lead signalling pathways in which DHX36 may be involved in lung cancer. Future studies should be encouraged to pin-point the actual cellular and molecular mechanisms of DHX36 function in lung cancer by either activation or overexpression of DHX36, which could be proceeded by either CRISPR-based genomic editing or small molecules predicted by chemical docking simulation. It is possible to better verify the functions of DHX36 by the combination of two-way regulation of this molecule or even ideally in an inducible manner. The possible discovery of DHX36 targeting may be translated to the treatment of lung cancer starting from an appropriate *in vivo* pre-clinical model.

## Conclusion

In this study, we show that DHX36 can be an independent prognosis indicator in NSCLC with subtype dependence. The stable knockdown of this helicase leads to promoted tumour growth, under 3D cultivation conditions and augmented cell colony formation, following single-cell cultivation. The DHX36 knockdown accelerates migration and aggregation of the S-phase subpopulation in lung cancer cells. Furthermore, the reduction of DHX36 level de-sensitises the proliferation response of lung cancer cells to chemotherapeutic drugs such as paclitaxel, with cell dependence. DHX36 functions through regulating multiple signalling pathways including activation of protein activity, protein autophosphorylation, Fc-receptor signalling pathway, response to peptide hormone and stress-activated protein kinase signalling cascade in lung cancer cells. A causal transcriptomic analysis suggests that DHX36 is significantly associated with mRNA surveillance, RNA degradation, DNA replication and Myc targets. Therefore, we unveil that DHX36 presents clinical significance and plays a role in tumour suppression in lung cancer, and propose a potentially new concept for an anti-cancer therapy based on helicase-specific targeting.

## Data Availability Statement

The original contributions presented in the study are included in the article/**Supplementary Material**. Further inquiries can be directed to the corresponding author.

## Ethics Statement

The study was approved by local ethics committees (Peking University Cancer Hospital) and performed in accordance with guidelines established by the World Medical Association Declaration of Helsinki. Written consent was obtained from all patients.

## Author Contributions

YC conceived, designed, and led this study. YC drafted the original manuscript. WJ contributed to the analysis of the Kinex antibody array data. YC provided bioinformatic and statistical analysis. ZL, JC, LZ, and XD contributed to other data analysis. JL and EB contributed to manuscript editing. All authors contributed to the article and approved the submitted version.

## Funding

This work was financially supported by grants from Cancer Research Wales, Life Sciences Research Network Wales and the Realcan Fellowship.

## Conflict of Interest

The authors declare that the research was conducted in the absence of any commercial or financial relationships that could be construed as a potential conflict of interest.

## Supplementary Material

Supplementary Figure 1 Pan-cancer analysis of DHX36 gene expression. **(A)** Analysis using the GEPIA bioinformatic server. **(B)** Analysis using the cBioPortal for Cancer Genomics.

Supplementary Figure 2 Additional analysis of the gene and protein expression levels of DHX36 in lung cancer. **(A)** The breakdown meta-analysis of the differential expression of DHX36 gene in lung cancer tissues using the Lung Cancer Explorer (http://lce.biohpc.swmed.edu). **(B)** Paired comparison of DHX36 gene expression in a gene microarray study (GSE18842). **(C)** The analysis of the DHX36 protein levels in lung cancer using the Clinical Proteomic Tumor Analysis Consortium (CPTAC).

Supplementary Figure 3 Initial cytotoxicity assay of the cell lines in response to cisplatin for the determination of the optimum dose for apoptosis detection. **(A)** Raw fluorescence reads of viable cells indicated by the Alamar Blue assay. **(B)** Normalised cell viability ratio.
