# Supplementary figures and images for: The G4 Resolvase DHX36 Possesses a Prognosis Significance and Exerts Tumour Suppressing Function Through Multiple Causal Regulations in Non-Small Cell Lung Cancer
Source: Front Oncol. 2021 Apr 27;11:655757. doi: 10.3389/fonc.2021.655757 (PMC8111079; doi:10.3389/fonc.2021.655757)

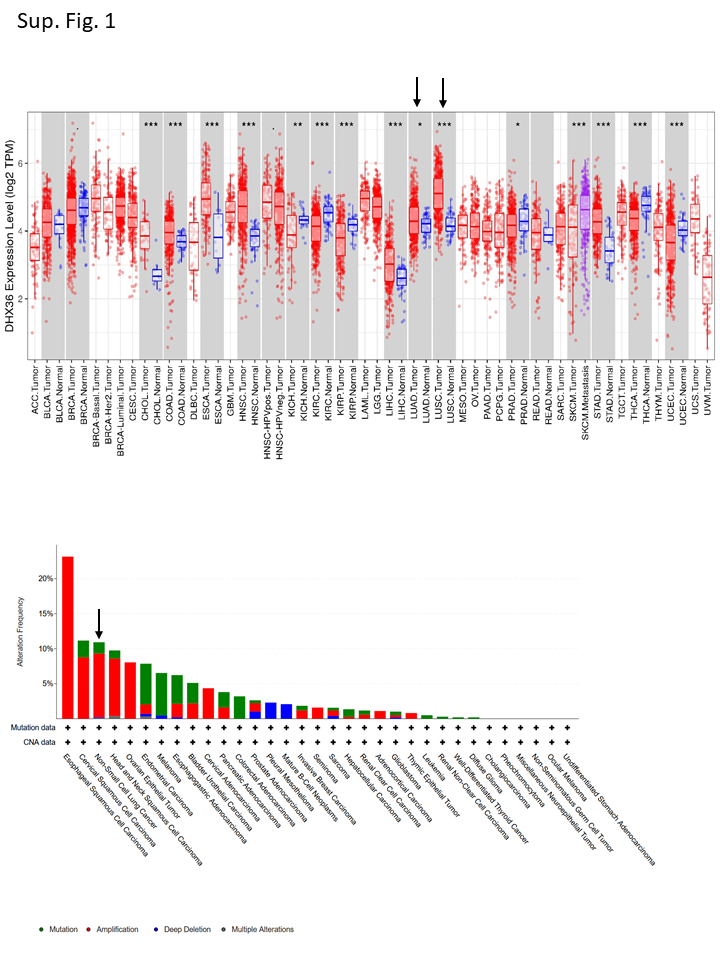

Supplement: Supplementary Figure 1 — Pan-cancer analysis of DHX36 gene expression. (A) Analysis using the GEPIA bioinformatic server. (B) Analysis using the cBioPortal for Cancer Genomics. [file Image_1.tif]

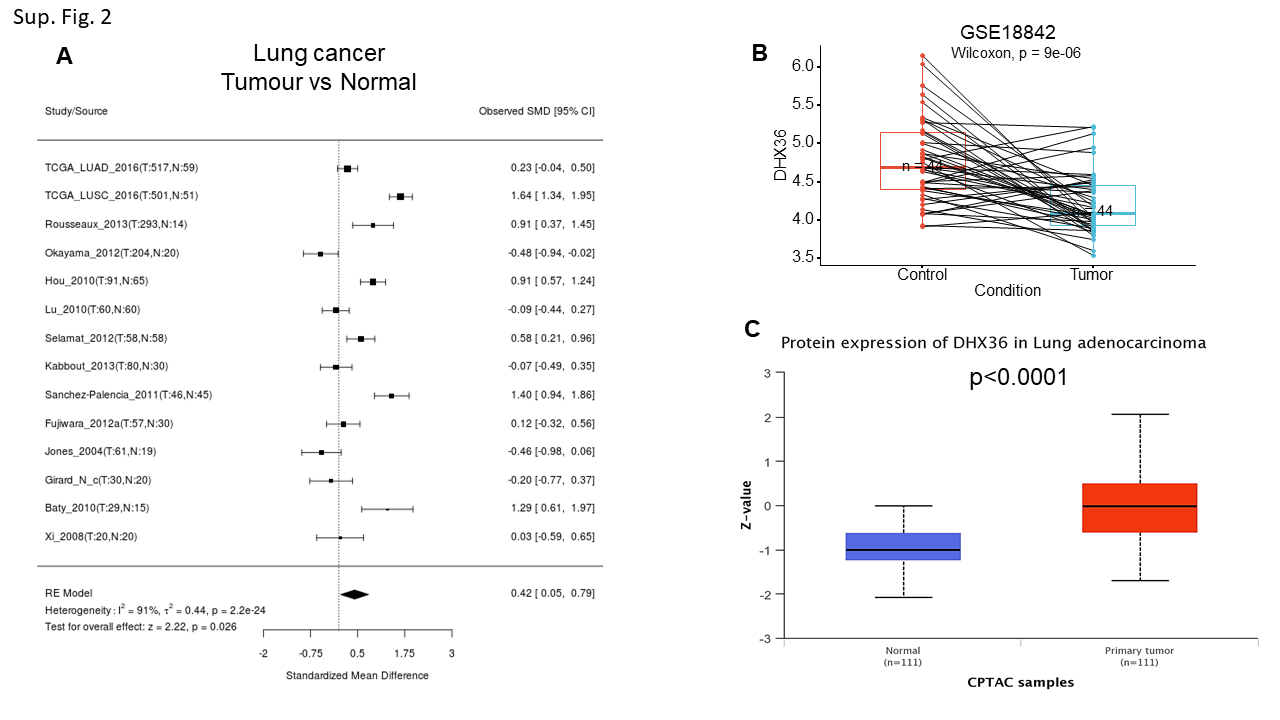

Supplement: Supplementary Figure 2 — Additional analysis of the gene and protein expression levels of DHX36 in lung cancer. (A) The breakdown meta-analysis of the differential expression of DHX36 gene in lung cancer tissues using the Lung Cancer Explorer (http://lce.biohpc.swmed.edu). (B) Paired comparison of DHX36 gene expression in a gene microarray study (GSE18842). (C) The analysis of the DHX36 protein levels in lung cancer using the Clinical Proteomic Tumor Analysis Consortium (CPTAC). [file Image_2.tif]

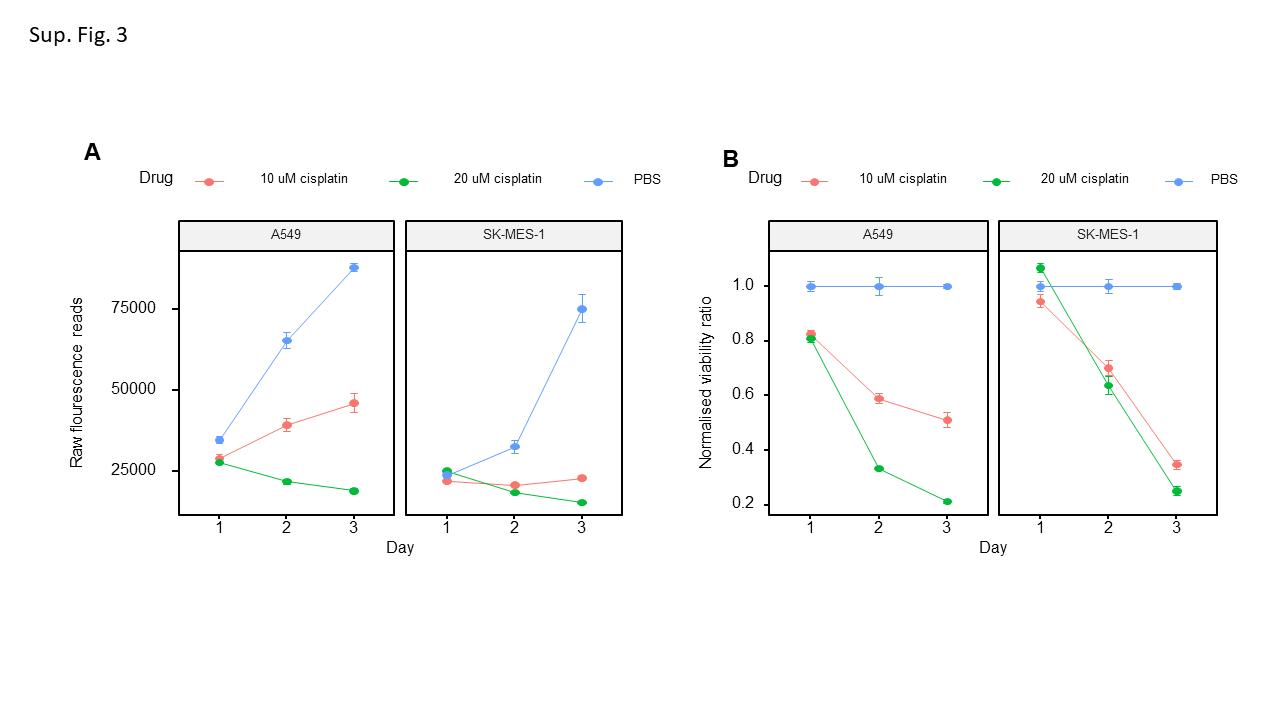

Supplement: Supplementary Figure 3 — Initial cytotoxicity assay of the cell lines in response to cisplatin for the determination of the optimum dose for apoptosis detection. (A) Raw fluorescence reads of viable cells indicated by the Alamar Blue assay. (B) Normalised cell viability ratio. [file Image_3.tif]
