# Supplementary material for: The G4 Resolvase DHX36 Possesses a Prognosis Significance and Exerts Tumour Suppressing Function Through Multiple Causal Regulations in Non-Small Cell Lung Cancer
Source: Front Oncol. 2021 Apr 27;11:655757. doi: 10.3389/fonc.2021.655757 (PMC8111079; doi:10.3389/fonc.2021.655757)
Supplement: Supplementary file 4 [file Table_1.docx]

**Supplementary Table 1. Transcript profiling of DHX36 in a lung cancer cohort by qRT-PCR.**

| **Clinicopathological feature** | | **N** | **Median** | **Min** | **Max** | **Q1** | **Q3** | **P value** |
| --- | --- | --- | --- | --- | --- | --- | --- | --- |
| Tissue type | Tumour | 58 | 1 | 0 | 8717 | 0 | 96 |  |
|  | Normal | 58 | 4 | 0 | 81368 | 0 | 277 | **p<0.05** |
| Histological Type | Squamous | 19 | 1 | 0 | 6454 | 0 | 127 |  |
|  | Adenocarcinoma. | 27 | 0.9 | 0 | 2379.9 | 0 | 60.6 | p>0.05 |
|  | others | 12 | 40 | 0 | 8717 | 0 | 4607 |  |
| Degree of differentiation | High | 6 | 1 | 0 | 2380 | 0 | 597 |  |
|  | High/Moderate | 11 | 2.8 | 0 | 578.3 | 0.6 | 74.3 | p>0.05 |
|  | Moderate | 13 | 36 | 0 | 6454 | 0 | 171 |  |
|  | Low | 8 | 0.9 | 0 | 89 | 0 | 84.3 |  |
| T-Staging | T-1 | 11 | 1 | 0 | 8717 | 0 | 213 |  |
|  | T-2 | 13 | 2 | 0 | 2380 | 0 | 233 |  |
|  | T-3 | 16 | 0.4 | 0 | 182.5 | 0 | 16.9 | p>0.05 |
|  | T-4 | 4 | 83.3 | 4.7 | 153.8 | 15.4 | 145.1 |  |
| Lymph node involvement | N-0 | 32 | 3 | 0 | 8717 | 0 | 183 |  |
|  | N-1 | 7 | 0.6 | 0 | 89 | 0 | 31.1 | p>0.05 |
|  | N-2 | 14 | 0 | 0 | 182.5 | 0 | 118.9 |  |
| TNM staging | TNM1 | 14 | 70 | 0 | 8717 | 0 | 1479 |  |
|  | TNM2 | 12 | 0.43 | 0 | 88.97 | 0.01 | 12.22 |  |
|  | TNM3 | 17 | 0.7 | 0 | 182.5 | 0 | 110.3 | p>0.05 |
|  | TNM4 | 1 | 47.772 | 47.772 | 47.772 |  |  |  |
| Smoking history (years) | Nonsmoker | 34 | 1 | 0 | 8717 | 0 | 54 |  |
|  | <10 | 2 | 0.626 | 0.264 | 0.988 |  |  |  |
|  | <20 | 5 | 4.7 | 0 | 280.9 | 1.7 | 156 |  |
|  | <30 | 10 | 103.9 | 0.1 | 213 | 10.3 | 175.3 | p>0.05 |
|  | <40 | 7 | 0 | 0 | 6454 | 0 | 578 |  |
|  | Life-time smoker | 24 | 18 | 0 | 6454 | 0 | 161 |  |
